# Conversion of partial restorer Swarna into restorer by transferring fertility restorer *Rf* gene(s) through marker assisted back cross breeding (MABB) in rice

**DOI:** 10.1038/s41598-020-58019-1

**Published:** 2020-01-24

**Authors:** Revathi Ponnuswamy, Arun Kumar Singh, Meenakshi Sundaram Raman, Lella venkata Subbarao, Neeraja C.N.

**Affiliations:** Crop Improvement Section, ICAR-Indian Institute of Rice Research, Rajendranagar, Hyderabad 500030 India

**Keywords:** Plant biotechnology, Plant breeding

## Abstract

The major constraints in hybrid rice breeding are availability of limited number of parental lines with specific desirable traits and lower frequency of restorers among elite breeding lines. The popular, high-yielding mega-rice variety Swarna, has been identified to be a partial restorer (as it has only one of major fertility restorer genes, *Rf4*) and hence cannot be utilized directly in the hybrid rice breeding. To convert the partial restorer to complete restorer, a cross was made between Swarna and a stable restorer KMR3R possessing *Rf*3 and *Rf*4 genes and developed BC_1_F_5_ and BC_2_F_4_ populations by marker-assisted back cross breeding (MABB). The SSR marker DRRM-RF3-10 linked to *Rf*3 gene located on chromosome 1, clearly distinguished restorers from partial restorers. All the improved lines of Swarna possessing *Rf3* and *Rf4* genes showed complete fertility restoration in test crosses with higher grain yield heterosis. Few rice hybrids developed by using converted restorers were evaluated in multi location testing under the All India Co-ordinated Rice Improvement Project (AICRIP). The results indicated that new rice hybrids expressed higher heterosis with matching grain quality attributes like Swarna. This study provides significantly novel and relevant restorers to enhance and economize future hybrid rice breeding programs.

## Introduction

Rice (*O. sativa* L.) is the most important staple food crop for more than half of the world’s population and it is cultivated in an area of 44.5 million hectare in India with the production of 106.5 million tonnes during the year 2016^1^. After the advent of high yielding semi-dwarf rice varieties, hybrid rice technology has been touted as a major strategy for enhancing the genetic yield potential of rice. The success of hybrid rice technology has been very well demonstrated in China, which produces 146.5 million tonnes of rice from 30.32 million hectares^1^. This significant increase in production in China is mainly due to cultivation of hybrid rice (with >50% area and production under rice hybrids). Several technical challenges, market and policy constraints has limited the development and diffusion of hybrid rice outside China^2^. In India, hybrid rice is cultivated in an area of ~3 million hectares^3^, which is about 6.7% of total area of rice cultivation. Hybrid rice accounts for less than 10% of the area under rice cultivation in Bangladesh, Indonesia, and the Philippines and just 10% in Vietnam.

Hybrid rice technology aims to increase the yield potential of rice by exploiting the phenomenon of hybrid vigour or heterosis. Cytoplasmic male sterility coupled with fertility restoration controlled by nuclear genes is a very useful tool in exploiting heterosis in self pollinated crops. In rice, three CMS systems viz. Wild Abortive (WA), Boro II (BT) and Honglian (HL) are deployed for commercial hybrid rice seed production^4,5^. The most widely used CMS system in rice is based on wild abortive (WA) cytoplasm derived from *Oryza sativa f. Spontanea*^6,7^. The WA-CMS system is highly stable with complete pollen sterility^8^. Hybrid rice seed production using CGMS/three line system involves a CMS (A) line, a maintainer (B) line and a restorer (R) line carrying the fertility restorer genes.

The restorers with strong restoration ability have two major genes along with modifier genes and a restorer with semi-restoring ability have either one of the two major genes^7^. Rice fertility restoration is governed by two independent dominant genes and one of the genes appeared to be stronger in action^9^ than the other. Bharaj *et al*.^10^ reported that fertility restoration is governed by two dominant genes, the restorers with dominant alleles of the two genes in homozygous or heterozygous condition will be fully fertile and the genotypes having dominant alleles of one of the two genes in homozygous or heterozygous condition but homozygous recessive alleles of the other gene will behave partially sterile or partially fertile and vice-versa.

The major loci restoring the fertility has designated as *Rf*3 and *Rf*4 and have been mapped on chromosome 1 and 10 using Zhenshan 97A near-isogenic lines (NILs) mapping population^11,12^. The chromosomal location of *Rf* genes has been resolved using RFLP (Restriction Fragment Length Polymorphism) markers and it showed that effect of the locus on chromosome 10 is larger than chromosome 1^13^. Mapping of two *Rf* genes of WA-CMS system on the long and short arm of chromosome 10 using SSLP (Simple Sequence Length Polymorphism) markers was done by Jing *et al*.^14^. The SSR primer RM258 located on chromosome 10 was found linked to the restorer gene at a distance of 9.5 cM^15^. Fertility restorer gene linked to RM6100 was mapped at a distance of 6–7 cM on chromosome 10 in the restorer lines viz., PRR 78 R, IR 40750 and MTU 9992^16^. The candidate gene based marker, DRRM-RF3-10 associated with *Rf3* locus showed maximum selection accuracy in identifying restores in comparison with other reported markers viz., RM10305, RM10318, DRRM-RF3-5 and DRRM-RF3-6^17^.

The process of screening for the trait of fertility restoration involves test crossing with a set of CMS lines and evaluation of hybrids/F_1_ for their pollen and spikelet fertility. According to the male fertility of F_1_ plants, the test lines can be classified as maintainers, restorers, partial restorers and partial maintainers^9^. Molecular markers linked to the fertility restoration trait are useful for evaluation of large number of germplasm/breeding lines to identify restorers in rice without progeny testing^18^. Based on molecular screening with markers linked to *Rf*4 and *Rf*3 genes^19^ reports that lines possessing both the *Rf* genes showed higher fertility than the genotypes containing *Rf3* or *Rf*4 individually. In hybrid rice breeding, elite lines and released varieties from varietal improvement programme are being tested for their fertility restoration ability by crossing with the CMS lines to identify restorers and maintainers. The restorer frequency among *indica* lines is only 40%^20^. The majority of the popular, mega varieties released in India like Swarna, Samba Mahsuri, and MTU1010 were found to be partial or incomplete restorers and hence cannot be utilized as such to produce experimental hybrids.

The major issues in hybrid rice breeding are lack of parental lines with desirable specific traits and lower frequency of restorers and maintainers among elite breeding lines. One of the major constraints, which limit the spread of hybrid rice area in shallow low lands and coastal areas, is non availability of long duration hybrids, which can mature in 145–150 days or more. The popular mega rice variety, Swarna has been widely adopted by farmers for its high yielding ability and adaption under low input conditions, shallow low lands. It is cultivated in an approximate area of 4.5 M ha^21^, occupying more than 30% of total rice area in eastern India^22^. Swarna possesses good grain and cooking quality with desirable grain type. However, as mentioned earlier, it cannot be utilized in hybrid breeding as it is a partial restorer. Therefore in the present investigation, an attempt was made to convert the partial restorer Swarna to a complete restorer by transferring major fertility restorer gene(s) through marker-assisted back cross breeding (MABB), and demonstrated the complete fertility restoration ability of the improved lines and developed new rice hybrids with attributes like Swarna.

## Results

### Fertility restoration status of parents

**Figure 1:**
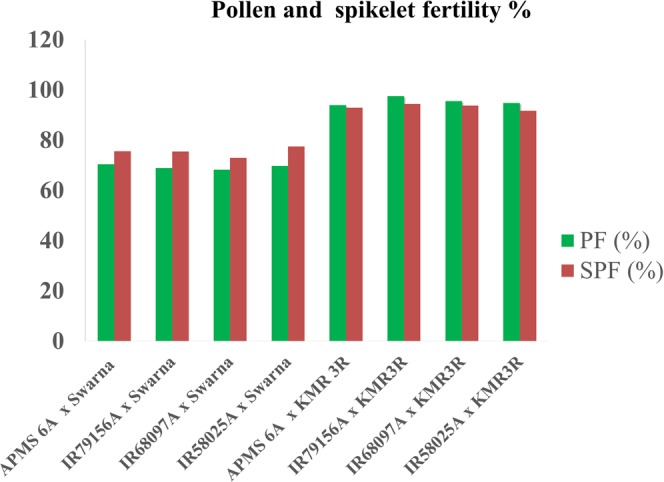
Pollen and spikelet fertility percentage of Swarna & KMR3R crossed with different CMS lines.

To study the fertility restoration status of donor and recurrent parents, F_1_ hybrids were produced by crossing Swarna and KMR3R with four CMS lines viz., APMS 6A, IR79156A, IR68897A and IR58025A. The F_1_ hybrids were evaluated for their pollen and spikelet fertility during both *kharif* (wet) and *rabi* (dry) seasons (Table 1). The average pollen and spikelet fertility percentage of partial restorer Swarna was observed to be less than 80%, whereas KMR3R showed more than 90% fertility restoration when crossed with different A lines (Fig. 1).

**Table 1:** Evaluation of pollen, and spikelet fertility percentage of F_1s_/hybrids.

A X R/PR cross	Pollen Fertility (%)		Spikelet Fertility (%)		Average	
	*Rabi*	*Kharif*	*Rabi*	*Kharif*	PF (%)	SPF (%)
APMS 6A × Swarna	69.3	71.5	74.3	76.9	70.4	75.6
IR 79156A × Swarna	68	69.8	73.5	77.5	68.9	77.5
IR 68097A × Swarna	67	69.5	72.3	73.7	68.3	73
IR 58025A × Swarna	69	70.5	76.5	78.4	69.8	77.5
APMS 6A × KMR3R	93	94.8	91.5	94.4	93.9	92.9
IR 79156A × KMR3R	95	100	96.5	92.5	97.5	94.5
IR 68097A × KMR3R	95	96	94.3	93.2	95.5	93.8
IR 58025A × KMR3R	97	92.5	90.3	93.1	94.8	91.7
Mean	81.66	83.08	83.65	84.96	82.39	84.56
SD	14.31	13.80	10.38	9.03	13.99	9.40
CV%	17.53	16.61	12.41	10.63	16.98	11.11

^*^A-CMS line, R- Restorer, PR- Partial restorer, PF- Pollen fertility, SPF- Spikelet fertility, SD-Standard deviation, CV- Co-efficient of variation.

### Molecular screening for the presence of fertility restorer *Rf* gene(s) with the help of reported markers

**Figure 2:**
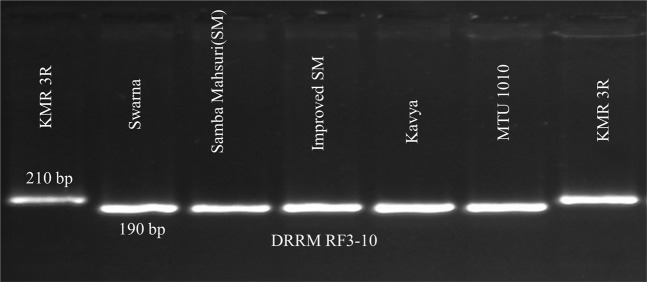
Amplification pattern of SSR marker DRRM-Rf3-10 between restorer (KMR3R) and partial restorers.

The popular restorer KMR3R and the variety Swarna were screened with the help of 20 reported SSR markers for the presence of fertility restorer gene *Rf*4 located on chromosome 10 and *Rf3* on chromosome 1(Table 2). The SSR markers DRRM-RF3-10 linked to *Rf3* gene clearly distinguished partial restorers from restorer KMR3R (Fig. 2). Interestingly Swarna showed the presence of *Rf*4 gene with respect to all the nine markers reported viz., RM6100, PPR3, RM 1108, RM 474, RM 311, RM 258, RM 591, DRCG-RF4-10 and DRCG-RF4-14. Therefore in the present study, we aimed to transfer the fertility restorer gene *Rf3* from KMR3R to Swarna by marker-assisted backcross breeding to develop complete restorers in the background of Swarna for utilizing in hybrid rice breeding programme.

**Table 2:** SSR markers linked to *Rf*_*4*_ & *Rf*_*3*_ genes of WA-CMS system.

Markers	Chromosome	Map distance	Linked gene	Reference
DRCG RF-4–10	10	5.5 cM	*Rf*_*4*_	Balaji *et al*., 2012^17^
DRCG RF-4–14	10	1.0 cM	*Rf*_*4*_	Balaji *et al*., 2012^17^
PPR 3 (pentatricopeptide repeat)	10	0.0	*Rf*_*4*_	Ngangkham *et al*., 2010^46^
RM 6100	10	6.7 cM	*Rf*_*4*_	Singh *et al*., 2005^16^
RM 1108	10	1.6 cM	*Rf*_*4*_	Sattari *et al*., 2008^35^
RM 474	10	1.2 cM	*Rf*_*4*_	Sheeba *et al*., 2009^36^
RM 311	10	1.2 cM	*Rf*_*4*_	Sheeba *et al*., 2009^36^
RM 258	10	3.1 cM	*Rf*_*4*_	Nematzadeh *et al*., 2010^47^
RM 591	10	31.3 cM	*Rf*_*4*_	Nematzadeh *et al*., 2010^47^
DRRM Rf-3–6	1	3.1 cM	*Rf*_*3*_	Balaji *et al*., 2012^17^
DRRM Rf-3–10	1	1.0 cM	*Rf*_*3*_	Balaji *et al*., 2012^17^
DRRM Rf-3–5	1	3.5 cM	*Rf*_*3*_	Balaji *et al*., 2012^17^
RM 10313	1	4.2 cM	*Rf*_*3*_	Neeraja *et al*., 2009^48^
RM 576	1	2.1 cM	*Rf*_*3*_	Sattari *et al*., 2008^35^
RM 315	1	20.7	*Rf*_*3*_	Bazrkar *et al*., 2008^49^
RM 7466	1	1.9 cM	*Rf*_*3*_	Bazrkar *et al*., 2008^49^
RM 443	1	4.4 cM	*Rf*_*3*_	Bazrkar *et al*., 2008^49^
RM 1	1	5.1 cM	*Rf*_*3*_	Alavi *et al*., 2009^50^
RM 3783	1	14 cM	*Rf*_*3*_	Alavi *et al*., 2009^50^
RM 3148	1	19.7	*Rf*_*3*_	Nematzadeh *et al*., 2010^47^

### Identification of SSR markers which are polymorphic between Swarna and KMR3R and details of background selection

**Figure 3:**
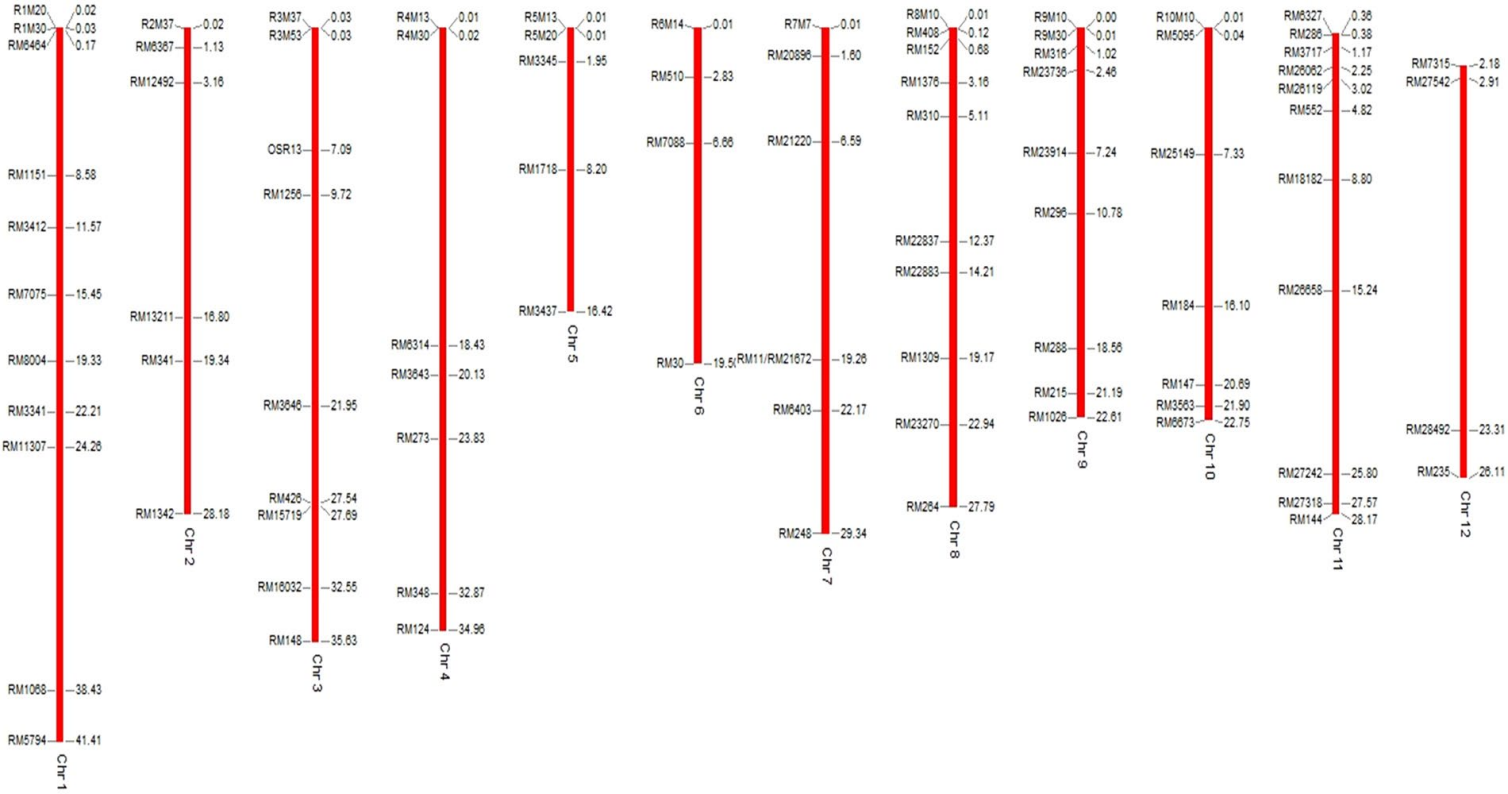
Chromosomal location of the polymorphic markers used for the background selection.

A set of 728 hyper-variable SSR markers were utilized for surveying polymorphism between Swarna and KMR3R across the 12 linkage groups of rice. Of these, 89 were polymorphic between the parents (12.22% polymorphism). In this study, chromosome 8 and 6 recorded the highest and lowest polymorphism percentage of 19.60% and 5.88%, respectively (Table 3). The chromosomal location map of polymorphic markers was prepared by graphical genotypes (GGT ver. 2.0) and same has been presented in the Fig. 3. In BC_1_F_1_ plants, the background recovery percentage ranged from 56.17 to 80% (data not shown) and seven plants were selected based on genome recovery. The chromosome 4, 7, 8 and 11 were shown complete recurrent parent type polymorphism, whereas the remaining allele ranged from 20% (Chromosome 5) to 55% (Chromosome 3). In BC_2_F_1_, out of 22 plants, three plants were selected based on foreground and background selection, which had shown complete genome recovery on chromosome 2, 3, 5 and 6 with 91% of recovery of Swarna genome.

**Table 3:** Polymorphic markers used for background selection of Swarna.

Chromosomal location	No. of markers utilized for screening	No. of identified Polymorphic markers	Polymorphic markers
1	85	11	R1M20, R1M30, RM6464, RM1151, RM3412, RM7075, RM8004, RM3341, RM11307, RM1068 & RM5794
2	65	6	R2M37, RM6367, RM12492, RM13211, RM341, & RM 1342
3	60	9	R3M37, R3M53, ORS13, RM1256, RM3646, RM426, RM15719, RM16032, & RM 148
4	55	7	R4M13, R4M30, RM6314, RM3643, RM273, RM348 & RM124
5	52	5	R5M13, R5M20, RM3345, RM1781 & RM3437
6	68	4	R6M14, RM510, RM7088 & RM30
7	68	6	R7M7, RM20896, RM21220, RM11/RM21672, RM6403 & RM248
8	51	10	R8M10, RM408, RM152, RM1376, RM310, RM22837, RM22883, RM1309, RM23270 & RM264
9	48	9	R9M10, R9M30, RM316, RM23736, RM23914, RM296, RM288, RM215 & RM1026
10	72	7	R10M10, RM5059, RM25149, RM184, RM147, RM3563 & RM6673
11	57	11	RM6327, RM286, RM3717, RM26062, RM26119, RM552, RM18182, RM26658, RM27242, RM27318 & RM144
12	47	4	RM7315, RM27542, RM28492 & RM235
Total no. of markers	728	89

### Marker assisted transfer of *Rf*_*3*_ gene into a partial restorer Swarna

Swarna was crossed with KMR 3R to produce F_1_ hybrids and F_1_ plants were confirmed for their heterozygosity with the help of SSR marker DRRM RF3-10 for the *Rf3* gene. The identified four “true” F_1_ plants were backcrossed with Swarna to produce BC_1_F_1_ seeds. Based on foreground selection, 41 BC_1_F_1_ plants were identified to be heterozygous for the presence of *Rf3* gene. Out of 41 plants, seven were identified through background selection. In the backcross generations the phenotypic traits viz., flowering duration, plant height, number of productive tillers and grain yield/plant were recorded and plants were visually selected for heavy pollen load with longer anther filament during anthesis, strong culm with specific features of Swarna like brown glume colour, stay greenness with desirable medium slender grains and were advanced to next generation. More emphasis was given to phenotypic selection for advancing plants to next generation along with background selection.

**Figure 4:**
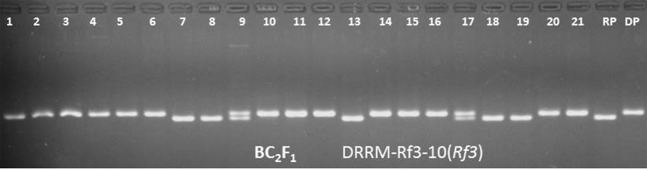
Selection of homozygous plants for the presence of *Rf3* gene in BC_2_F_1_ population. (*Rf3* gene 210 bp presence 1 to 6, 10–12, 14–16, 20, 21), RP -recurrent parent (Swarna), DP-Donor parent (KMR3R).

One superior BC_1_F_1_ plant with maximum recurrent parent genome (80%) along with better agro morphological traits (i.e. similar to Swarna) was backcrossed with Swarna to produce BC_2_F_1_ seeds. Simultaneously, the selected BC_1_F_1_ plant with maximum recurrent parent genome was selfed to produce BC_1_F_2_ seeds. By foreground selection in BC_2_F_1_ and BC_1_F_2_ generation plants carrying *Rf3* gene in homozygous and heterozygous conditions were identified (Fig. 4). A total 65 plants (22 BC_2_F_1_, 43 BC_1_F_2_) possessing superior phenotypes were subjected to background selection analysis. BC_2_F_1_ and BC_1_F_2_ plants with maximum recurrent parent genome (RPG) were selfed to produce BC_2_F_2_ and BC_1_F_3_ seeds. The BC_2_F_2_ and BC_1_F_3_ plants were raised and subjected to foreground selection for the presence of *Rf3* gene. Marker-assisted screening resulted in identification of 35 plants to carry *Rf3* in homozygous condition in BC_2_F_2_ and 40 plants in BC_1_F_3_ generation. The identified homozygous plants were selfed to raise BC_2_F_3_ and BC_1_F_4_ generations. Phenotypically superior plants were identified based on morphological traits at BC_2_F_3_ and BC_1_F_4_ generations and were selfed to produce the subsequent generation seeds. BC_2_F_4_ and BC_1_F_5_ lines were evaluated in three rows and plants were subjected to stringent phenotypic selection and agro morphological evaluation.

### Evaluation of the improved lines of BC_2_F_4_ and BC_1_F_5_ for Agro morphological traits

**Figure 5:**
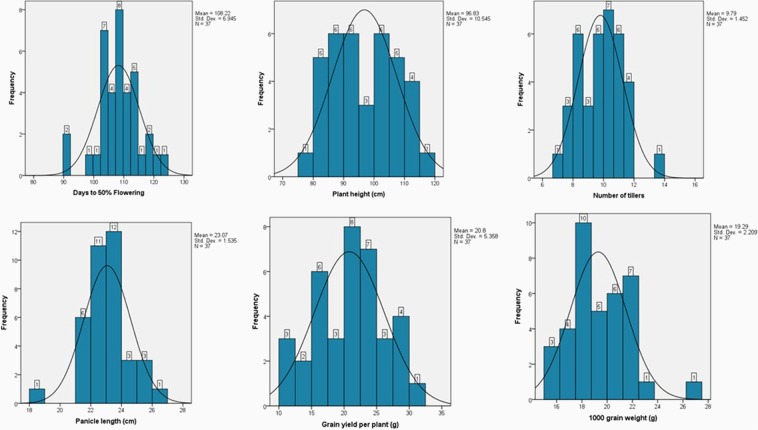
Frequency distribution of yield related traits in BC_2_F_4_ and BC_1_F_5_ generations of Swarna x KMR3R.

The improved lines of BC_2_F_4_ and BC_1_F_5_ generations derived from the cross Swarna x KMR3R, were evaluated in alpha lattice design along with parents and checks in 6 sq. m plot during *kharif*  2015 at Indian Institute of Rice Research, Rajendranagar, Hyderabad. The observations were recorded on five plants in each replication and mean data is presented in (Table 4) and frequency distribution of each traits are presented in (Fig. 5).

**Table 4:** Agro-morphological evaluation of converted restorer lines in the background of Swarna.

Restorer lines	DFF	PH (cm)	No. of productive tillers	PL (cm)	GY/P (g)	1000 Grain wt (g)
RP 5934–66	111.0 ± 1.0	93.9 ± 4.9	11 ± 0.4	21.4 ± 0.7	19.7 ± 0.3	19.2 ± 0.03
RP 5934–67	112.5 ± 0.5	112.8 ± 2.1	11 ± 0.2	22.7 ± 1.4	22.4 ± 1.4	21.4 ± 0.12
RP 5934–68	111.5 ± 1.5	113.7 ± 1	10 ± 0.5	24.9 ± 0.2**	16.2 ± 1.2	20.4 ± 0.1
RP 5934–69	98.5 ± 1.5	97.7 ± 0.6	11 ± 0.6	22.7 ± 0.3	23.1 ± 0.1	17.4 ± 0.07
RP 5934–70	103.5 ± 1.5	100.2 ± 1.9	10 ± 0.2	23.2 ± 0.2	25.1 ± 1.9	21.5 ± 0.1
RP 5934–71	105.0 ± 2.0	108.1 ± 1.9	12 ± 0.3**	23.1 ± 0.1	19.9 ± 1.1	19.1 ± 0.2
RP 5934–72	104.0 ± 1.0	102.7 ± 0.7	9 ± 1.1	23.4 ± 0.8	23.7 ± 1.3	20.0 ± 0.1
RP 5934–73	105.0 ± 2.0	102.9 ± 2.5	11 ± 0.7	24.8 ± 0.6**	26.2 ± 1.2	15.1 ± 0.1
RP 5934–74	103.5 ± 1.5	112.4 ± 0.8	10 ± 0.4	22.4 ± 0.1	24.4 ± 2.1	20.2 ± 0.1
RP 5934–75	102.5 ± 1.5	107.4 ± 1.1	9 ± 0.6	22.5 ± 0.5	20.5 ± 0.5	18.0 ± 0.1
RP 5934–76	102.5 ± 0.5	111.6 ± 2	10 ± 0.7	22.4 ± 0.9	29.4 ± 0.9	20.5 ± 0.1
RP 5934–77	105.0 ± 1.0	105.3 ± 3	8 ± 1.6	22.8 ± 0.5	20.9 ± 0.9	18.7 ± 0.1
RP 5934–78	107.5 ± 0.5	108.1 ± 2.1	11 ± 1.1	23.3 ± 1	16.0 ± 0.1	17.8 ± 0.2
RP 5934–79	108.0 ± 1.0	94.1 ± 0.2	9 ± 0.3	23.1 ± 0.5	20.5 ± 0.5	21.4 ± 0.1
RP 5934–80	105.5 ± 0.5	90.5 ± 0.1	8 ± 0.4	22.8 ± 0.2	20.5 ± 1.1	21.5 ± 0.1
RP 5934–81	113.5 ± 1.5	89.3 ± 1.8	11 ± 1.2	25.9 ± 0.2**	22.7 ± 0.5	19.2 ± 0.01
RP 5934–82	109.5 ± 0.5	97.8 ± 1.9	10 ± 0.7	24.9 ± 0.2**	30.5 ± 0.5**	22.6 ± 0.16
RP 5934–83	113.5 ± 0.5	91.7 ± 2.0	10 ± 0.1	23.4 ± 0.2	22.9 ± 2.2	18.2 ± 0.05
RP 5934–84	116.5 ± 0.5	97.7 ± 2.1	11 ± 0.3	23.1 ± 1.4	20.9 ± 4.1	19.35 ± 0.02
RP 5934–85	113.5 ± 0.5	102.6 ± 1.3	8 ± 2.5	23.3 ± 0.8	21.2 ± 3.8	20.1 ± 0.1
RP 5934–86	111.5 ± 0.5	107.6 ± 3.8	14 ± 1.5**	26.4 ± 0.7**	21.9 ± 1.9	21.5 ± 0.2
RP 5934–91	117.5 ± 0.5	102.9 ± 1.5	10 ± 0.2	22.5 ± 0.9	29.0 ± 6.0**	15.5 ± 0.04
RP 5934–100	104.0 ± 1.0	101.8 ± 3.8	8 ± 0.2	23.8 ± 0.2	29.0 ± 4.0**	17.4 ± 0.12
RP 5934–87	91.0 ± 1.0	82.7 ± 5.7	9 ± 0.9	21.7 ± 0.7	10.3 ± 0.3	18.2 ± 0.2
RP 5934–88	119.0 ± 1.0	92.4 ± 2.4	8 ± 0.6	25.6 ± 0.6**	10.8 ± 0.8	18.7 ± 0.13
RP 5934–89	114.5 ± 0.5	88.5 ± 4.5	8 ± 0.7	25.5 ± 0.8**	23.6 ± 2.2	19.1 ± 0.1
RP 5934–90	120.5 ± 0.5	79.6 ± 6.9	7 ± 0.7	21.2 ± 1.2	20.5 ± 1.2	17.2 ± 0.2
RP 5934–92	108.0 ± 2.0	85.2 ± 2.6	8 ± 0.9	22.2 ± 1.2	24.8 ± 4.8	21.3 ± 0.1
RP 5934–93	107.5 ± 0.5	81.1 ± 3.1	10 ± 0.2	21.0 ± 0.7	16.6 ± 3.4	18.1 ± 0.1
RP 5934–94	104.5 ± 0.5	89.1 ± 2.9	9 ± 1.4	21.5 ± 0.5	19.9 ± 1.8	20.1 ± 0.02
RP 5934–95	111.5 ± 0.5	84.5 ± 6.1	11 ± 0.6	22.7 ± 0.4	15.0 ± 5.0	18.5 ± 0.1
RP 5934–96	102.0 ± 1.0	86.8 ± 3.5	10 ± 1.3	22.9 ± 0.5	13.3 ± 1.8	17.6 ± 0.1
RP 5934–97	91.0 ± 1.0	90.2 ± 3.2	10 ± 0.5	23.7 ± 0.3	15.7 ± 0.7	21.9 ± 0.1
RP 5934–98	109.0 ± 1.0	86.8 ± 0.8	10 ± 0.2	23.5 ± 1.2	14.8 ± 4.8	17.4 ± 0.1
RP 5934–99	109.5 ± 1.5	84.3 ± 3.9	12 ± 2.9**	21.2 ± 0.6	25.6 ± 10.6	17.7 ± 0.1
KMR3R	108.5 ± 1.5	115.7 ± 5.3	11 ± 1.2	23.9 ± 0.1	27.6 ± 2.6	26.5 ± 0.2
Swarna	122.5 ± 2.5	83.1 ± 2.9	11 ± 1.2	18.52 ± 0.29	15.11 ± 0.10	15.85 ± 0.04
**F Value**	35.77	14.91	2.23	7.15	3.28	4.71
**p- value**	<0.001	<0.001	<0.001	<0.001	<0.001	<0.001
**CV (%)**	1.5	4.0	14.0	3.6	20.0	7.4
**LSD (0.05)**	3.3	7.8	2.8	1.7	8.4	2.9

^*^DFF- Days to 50% flowering; cm: centimeter; g: gram; ± Standard error, **Better than both the parents.

#-Data was recorded from five randomly selected plants of both replications.

Plant height of selected improved lines ranged from 79.6 cm to 113.7 cm. Swarna was 83.1 cm tall, whereas KMR3R possessed a plant height 115.7 cm. The improved lines possessing more than 100 cm plant height were preferred for using it as restorers, most of the lines possessed taller plant height as compared to recurrent parent Swarna, which is one of the most desirable trait for an ideal restorer parent for utilizing them in hybrid rice breeding. Number of productive tillers and panicle length in the improved lines ranged from 7 to 14 and 21 cm to 26.4 cm respectively, whereas both donor and recurrent parent had 11 productive tillers and possessed panicle length of 18.5 cm (Swarna) and 23.9 cm (KMR3R). The panicle length of converted restorer lines were significantly longer (>7 cm) than Swarna. With respect to mean grain yield per plant of selected backcross derived lines, ranged from 19.0 to 25.0 gm, which is significantly higher than the recurrent parent, Swarna (15.28 gm). The test weight of Swarna (1000 grain weight) was 15.9 gm with short bold grains, whereas KMR3R possessed 26.5 gm of test weight with long bold grain type. The converted restorer lines test weight ranged from 15.1 to 21.9 gm with the grain type of short slender to short bold. Days to 50% flowering (DFF) of selected backcross derived lines ranged from 91 to 121 days (Fig. 5), whereas Swarna and KMR3R  flowered on 123 and 109 days, respectively. Maximum number of improved lines were observed to flower as that of recurrent parent Swarna and few lines flowered significantly early i.e., 15–25 days than Swarna.

### Evaluation of experimental hybrids developed by utilizing converted improved restorer lines

The phenotypically superior lines of BC_2_F_4_ and BC_1_F_5_ generations were crossed with two CMS lines namely APMS 6A and CRMS 32A to develop experimental rice hybrids. The improved lines possessing both *Rf3* and *Rf*4 along with the maximum recovery of recurrent parent genome (RPG) and possessing plant height of more than 100 cm were crossed with above mentioned CMS lines to produce F_1_ hybrids. F_1_ hybrids were evaluated for pollen and spikelet fertility percentage and grain yield heterosis along with different duration checks. Based on days to 50% flowering three hybrid groups *viz*., early (<100 days), medium (101–110 days) and late (>110 days) were constituted for evaluation. (Table 5a–c).

**Table 5:** Evaluation of early duration F_1_/Hybrids developed utilizing improved Swarna restorers.

F_1_/Hybrids	DFF	PH (cm)	Tillers	PL (cm)	PF (%)	SPF (%)	GY/P (g)
**a**							
APMS 6A × RP 5934–69	98	110 ± 1.2	16 ± 0.6	23 ± 0.14	100 ± 0	85 ± 1.73	38 ± 1.16
APMS 6A × RP 5934–70	99	113 ± 1.2	10 ± 0.6	26 ± 0.72	95 ± 0	87 ± 1.73	42 ± 1.16
CRMS 32A × RP 5934–97	98	117 ± 1.7	8 ± 0.6	22 ± 0.29	100 ± 0	92 ± 1.15	35 ± 1.16
CRMS 32A × RP 5934–69	98	111 ± 1.2	9 ± 0.9	20 ± 0.58	100 ± 0	95 ± 0.58	37 ± 1.16
**Checks**							
US 314	95	106 ± 1.2	9 ± 0.6	20 ± 0.29	100 ± 0	95 ± 1.15	30 ± 1.16
DRRH 3	107	107 ± 1.2	10 ± 1.2	23 ± 0.58	95 ± 0	95 ± 1.15	25 ± 1.16
**b**							
APMS 6A × RP 5934–73	105	110 ± 1.15	12 ± 0.58	24 ± 0.58	73 ± 0	95 ± 1.15	55 ± 0.58
APMS 6A × RP 5934–75	105	110 ± 0.58	13 ± 0.58	24 ± 0.29	85 ± 0	90 ± 0.58	40 ± 1.15
APMS 6A × RP 5934–76	100	110 ± 1.15	15 ± 0.58	22 ± 0.58	87 ± 0	93 ± 0.87	45 ± 0.87
APMS 6A × RP 5934–82	106	108 ± 1.15	14 ± 0.58	24 ± 0.58	95 ± 0	87 ± 0.58	45 ± 0.29
APMS 6A × RP 5934–77	105	106 ± 0.58	11 ± 0.58	25 ± 0.29	100 ± 0	92 ± 0.58	45 ± 0.58
APMS 6A × RP 5934–78	103	107 ± 0.58	14 ± 0.58	22 ± 0.58	95 ± 0	93 ± 0.58	55 ± 1.15
APMS 6A × RP 5934–80	105	106 ± 0.29	9 ± 0.58	23 ± 0.58	85 ± 0	95 ± 0.58	55 ± 0.29
APMS 6A × RP 5934–66	105	102 ± 1.15	10 ± 0.58	25 ± 1.15	81 ± 0	89 ± 0.58	45 ± 0.87
APMS 6A × RP 5934–71	105	114 ± 1.15	11.5 ± 0.29	25 ± 0.58	85 ± 0	91 ± 0.58	50 ± 0.58
CRMS 32A × RP 5934–73	107	109 ± 0.58	9.5 ± 0.29	22 ± 0.58	90 ± 0	95 ± 0.58	45 ± 0.58
CRMS 32A × RP 5934–78	108	100 ± 1.15	8.5 ± 0.29	21 ± 0.58	80 ± 0	90 ± 0.58	41 ± 0.58
CRMS 32A × RP 5934–79	103	102 ± 0.58	9.5 ± 0.29	26 ± 0.58	82 ± 0	89 ± 0.58	46 ± 0.87
CRMS 32A × RP 5934–100	110	114 ± 1.15	15 ± 0.58	25 ± 0.29	81 ± 0	100 ± 0.29	50 ± 0.58
APMS 6A × Swarna	107	95 ± 1.15	9 ± 0.58	20 ± 0.58	70 ± 0	79 ± 0.58	19 ± 0.29
APMS 6A × KMR3R	103	121 ± 0.58	11.5 ± 0.87	23 ± 0.58	100 ± 0	85 ± 0.87	30 ± 0.58
CRMS 32A × Swarna	110	91 ± 0.87	10 ± 0.58	22 ± 0.58	65 ± 0	76 ± 0.29	20 ± 0.58
CRMS 32A × KMR3R	100	115 ± 0.58	12 ± 0.58	25 ± 0.58	100 ± 0	85 ± 0.58	30 ± 0.87
**Checks**							
HRI 174	103	106 ± 1.15	10 ± 0.58	22 ± 0.58	100 ± 0	92 ± 0.87	37 ± 0.58
DRRH 3	107	101 ± 0.58	7 ± 0.58	23 ± 0.29	100 ± 0	95 ± 0.58	25 ± 0.58
US 312	105	101 ± 0.87	7 ± 0.58	21 ± 0.58	100 ± 0	100 ± 0.29	35 ± 0.58
**c**							
APMS 6A × RP 5934–84	111	102 ± 0.58	13 ± 0.58	24 ± 0.58	100 ± 0	87 ± 1.15	30 ± 1.15
APMS 6A × RP 5934–67	111	105 ± 0.58	9 ± 0.58	24 ± 0.58	95 ± 1.15	85 ± 1.15	35 ± 1.15
APMS 6A × RP 5934–88	120	108 ± 0.58	9 ± 0.58	22 ± 0.58	95 ± 1.15	95 ± 1.15	30 ± 0.58
CRMS 32A × RP 5934–91	116	97 ± 0.87	9 ± 0.58	21 ± 0.58	97 ± 1.15	90 ± 2.89	38 ± 0.58
CRMS 32A × RP 5934–81	111	101 ± 0.58	10 ± 0.58	25 ± 0.58	80 ± 2.89	90 ± 1.15	45 ± 0.58
CRMS 32A × RP 5934–83	120	106 ± 0.58	11 ± 0.58	24 ± 0.58	90 ± 2.89	92 ± 1.15	37 ± 0.87
CRMS 32A × RP 5934–84	111	105 ± 0.58	8 ± 0.58	23 ± 0.58	100 ± 0	93 ± 0.58	35 ± 0
CRMS 32A × RP 5934–89	112	106 ± 1.15	10 ± 0.58	24 ± 0.58	100 ± 0	95 ± 1.15	42 ± 0.87
WGL 14	111	100 ± 0.58	11 ± 0.58	19 ± 0.58	—	—	30 ± 1.15
DRRH 3	107	101 ± 0.58	10 ± 0.58	23 ± 0.58	—	—	25 ± 0.58
Swarna	121	95 ± 1.15	12 ± 0.58	20 ± 0.58	—	—	19 ± 0.29

^*^DFF- Days to 50% flowering, PH- Plant height, cm- Centimeter, PL- Panicle length, PF- Pollen fertility, SPF- Spikelet fertility, GY/P- Grain yield per plant, g- gram, ± Standard error.

Evaluation of medium duration F_1_/Hybrids developed utilizing improved Swarna restorers.

Evaluation of late duration F_1_/Hybrids developed utilizing improved Swarna restorers.

### Early duration hybrids

In early duration hybrids, pollen and spikelet fertility percent ranged from 93.23 to 100% and 85 to 95.3%, respectively. Grain yield per plant ranged from 35 to 42 grams and plant height was measured to be 83.75 to 90.60 cm. The early hybrid US 314 was used as a check hybrid for estimating standard heterosis. It was found that restorer line RP5934-70 showed 40% standard heterosis. Hence this could be a potential restorer for developing early duration rice hybrids with characteristic features of Swarna (Table 5a).

### Medium duration hybrids

In medium duration hybrids, the pollen and spikelet fertility percent ranged from 82 to 100% and 85 to 95.3%, respectively. The single plant yield of hybrids ranged from 32 to 55 grams with the plant height of 83.75 to 90.60 cm. The medium duration hybrid HRI 174 was used as a check for estimating standard heterosis. It was found that improved restorer lines viz., RP5934-73, 78 and 80 lines showed more than 48% and RP5934-71 more than 35% of standard heterosis, while crossing with APMS 6A. The converted line RP 5934-100 showed a standard of heterosis of more than 35%, when it was crossed with CRMS 32A. These five restorers can be utilized for the developing medium duration hybrids with higher heterosis (Table 5b).

### Late duration hybrids

Eight late duration hybrids were evaluated for pollen and spikelet fertility, yield and yield related traits. The hybrids derived from improved lines viz., RP5934-81 and 89 showed a standard yield heterosis of more than 40%, when they were crossed with CRMS 32A. These restorers could be potential restorers for developing late duration rice hybrids (Table 5c).

### Multi-location evaluation of the newly derived hybrids

Based on the performance of converted improved restorer lines in the research station trial, two high yielding hybrids namely IIRRH-111 and IIRRH-112 were nominated for multi-location evaluation in AICRIP (All India Co-ordinated Rice improvement project)-IHRT trial during *kharif* 2016. In the case of AICRIP- hybrid rice network system, the experimental hybrids developed by public and private sectors were evaluated in Initial Hybrid Rice Trials (IHRT) in different zones in India. The hybrid entries with a yield superiority of more than 5% and 10% over the best hybrid and varietal checks either on over all mean basis or on zonal mean basis are promoted from IHRT to next stages of testing in Advance Varietal Trial 1 (AVT 1) and AVT 2^23^.

The rice hybrid IIRRH-112 showed a superior performance over the checks (AICRIP progress report, 2016) and registered yield superiority over checks during *kharif* 2016 multi-location testing. With respect to quality data collected from the AICRIP progress report (2016), this hybrid possessed short bold grains as that of Swarna and preferred intermediate amylose content (26.22%) with 60% high head rice recovery. In AICRIP testing rice hybrids were evaluated for not only yield and also for their grain quality characters. The grain quality characters of hybrids developed by crossing CMS lines with improved restorers are presented in Table 6. The improved restorers, which showed superior performance at IIRR station trial, were nominated for multi-location testing and are presently under AICRIP evaluation.

**Table 6:** Grain quality characteristics of hybrids developed utilizing improved restorers along with checks.

Entry	Hull (%)	Mill (%)	HRR	KL	KB	L/B	Grain type	Grain chalk	ASV	AC	GC
APMS 6A × RP 5934–73 (IIRRH 111)	79.8	66.1	47.9	5.76	1.91	3.1	SS	A	5	26.63	22
IR79156A × RP 5934–76	79.8	66.1	47.9	5.76	1.91	3.1	SS	VOC	5	26.63	22
APMS 6A × RP 5934–78	81.0	70.7	59.8	5.46	2.21	2.47	SB	VOC	4	26.22	41
APMS 6A × RP 5934–71	78.2	71.2	60.9	5.30	2.0	2.65	SB	VOC	5	25.32	22
APMS 6A × RP 5934–80	80.0	70.5	61.8	5.46	2.21	2.47	SB	VOC	5	24.67	41
CRMS 32A × RP 5934–100	79.8	66.1	47.9	5.76	1.91	3.1	SS	VOC	5	25.63	22
Swarna	77.8	67.9	62.1	5.21	2.22	2.34	SB	VOC	5	24.05	25
KRH 2	77.6	73.0	57.3	6.1	2.2	2.8	LB	VOC	2.2	27.0	22
DRRH 3	80.3	72.5	69.1	5.56	2.03	2.63	MS	A	5	24.52	24
Improved Samba Mahsuri	75.4	66.6	60.6	5.16	1.82	2.83	MS	VOC	5	23.78	35
Samba Mahsuri	79.5	69.9	65.2	5.23	1.85	2.82	MS	A	5	24.28	40

^*^Hull: Hulling (%), Mill: Milling %, HRR: Head rice recovery, KL: Kernal length (mm), KB: Kernal breadth (mm), SS: Short slender, L/B: Length and breadth ratio, VOC: Very occasionally present, A: Absent, ASV: Alkali spreading value, AC: Amylose content (%), GC: Gel consistency, SB: Short bold.

## Discussion

Hybrid rice technology is likely to play a pivotal role in increasing the rice production. Hybrid rice has been commercialized in countries like China, India, Vietnam, Philippines, Bangladesh, Indonesia, Pakistan, Ecuador, Guinea and the United States of America^24^. In India, as a result of concerted efforts by both public and private sectors, a total 102 hybrids have been released for commercial cultivation in the country. In spite of having great potential to enhance rice production and productivity, area expansion under hybrid rice has not increased significantly in the last few years due to major constraints like non availability of long duration hybrids suitable for shallow lowlands and coastal areas, poor grain and cooking quality, marginal heterosis, higher seed cost, non-involvement of public sectors in hybrid rice seed production^25^. The popular, high yielding, mega rice variety Swarna, with good grain and cooking quality, cannot be utilized as such in hybrid breeding due to their partial restoration of fertility of CMS lines (pollen and spikelet fertility is less than 80%) (Table 1). The effective restorers are expected to have more than 90% of pollen and spikelet fertility to develop new rice hybrids^26^. While considering the above stated constraints and wider scope for large scale adoption of rice hybrids, particularly the ones suited for rainfed shallow low lands and costal area, the present study was planned and carried out with an objective to develop restorers with good grain and cooking quality in the background of mega Indian rice variety Swarna, so that new hybrids can be developed for the long duration segment with better grain and cooking quality with higher level of heterosis.

Molecular screening for the presence of *Rf* genes with the reported SSR markers (Table 2) results indicated that partial restorer Swarna showed the presence of *Rf*4 gene and the absence of *Rf3* gene. The marker DRRM RF3-10 located on chromosome 1, could clearly differentiated restorers from partial restorers like Swarna, BPT 5204 (Samba Mahsuri) and Improved Samba Mahsuri, Kavya and MTU 1010 (Fig. 2) in our study. Hence we report here that SSR marker DRRM RF3-10 located on chromosome 1 is a very useful marker in distinguishing partial restorers from restorer and it may be useful in marker assisted selection (MAS) for the fertility restoration trait of WA-CMS system.

An earlier study^18^, reported that the stable restorer KMR3R possessing both the major fertility restorer genes *Rf*4 and *Rf3* and this was also confirmed in our marker analysis. Hence, KMR3R was utilized as a donor parent for *Rf* gene(s) particularly *Rf3* gene located on chromosome 1. Backcross breeding is the most commonly used method for incorporating any essential genes into a rice cultivar. Backcross breeding along with marker-assisted foreground and background selection contributes immensely to accelerate recurrent parent genome (RPG) recovery^27^.

Marker-assisted backcross breeding which includes MAS for the target gene(s) known as foreground selection and MAS for the recovery of recurrent parent genome known as background selection^28^ considered to be an efficient strategy for transferring targeted specific genes to elite lines. MABB is a most effective breeding strategy in rice for improving simple and complex traits. Some of the popular rice varieties released for cultivation in India through MAS/MABB for biotic and abiotic stress tolerance are Pusa Basmati 1 with bacterial blight (BB) resistance genes *xa13* and *Xa21* involving single back cross generation followed by selfing and pedigree based selection^29^ and Improved Samba Mahsuri with three BLB resistance genes viz., xa5, *xa13* and *Xa21* involving four back crossing^30^. Swarna Sub1 with *Sub1* QTL for the submergence tolerance^31^ and IR64 Drt 1 with two yield QTLs under drought situations namely *qDTY*_*2.2*_ and *qDTY*_*4.1*_ (IR64 NILs) for drought tolerance^32^ are the best examples of abiotic stress tolerance varieties derived through MAS.

**Figure 6:**
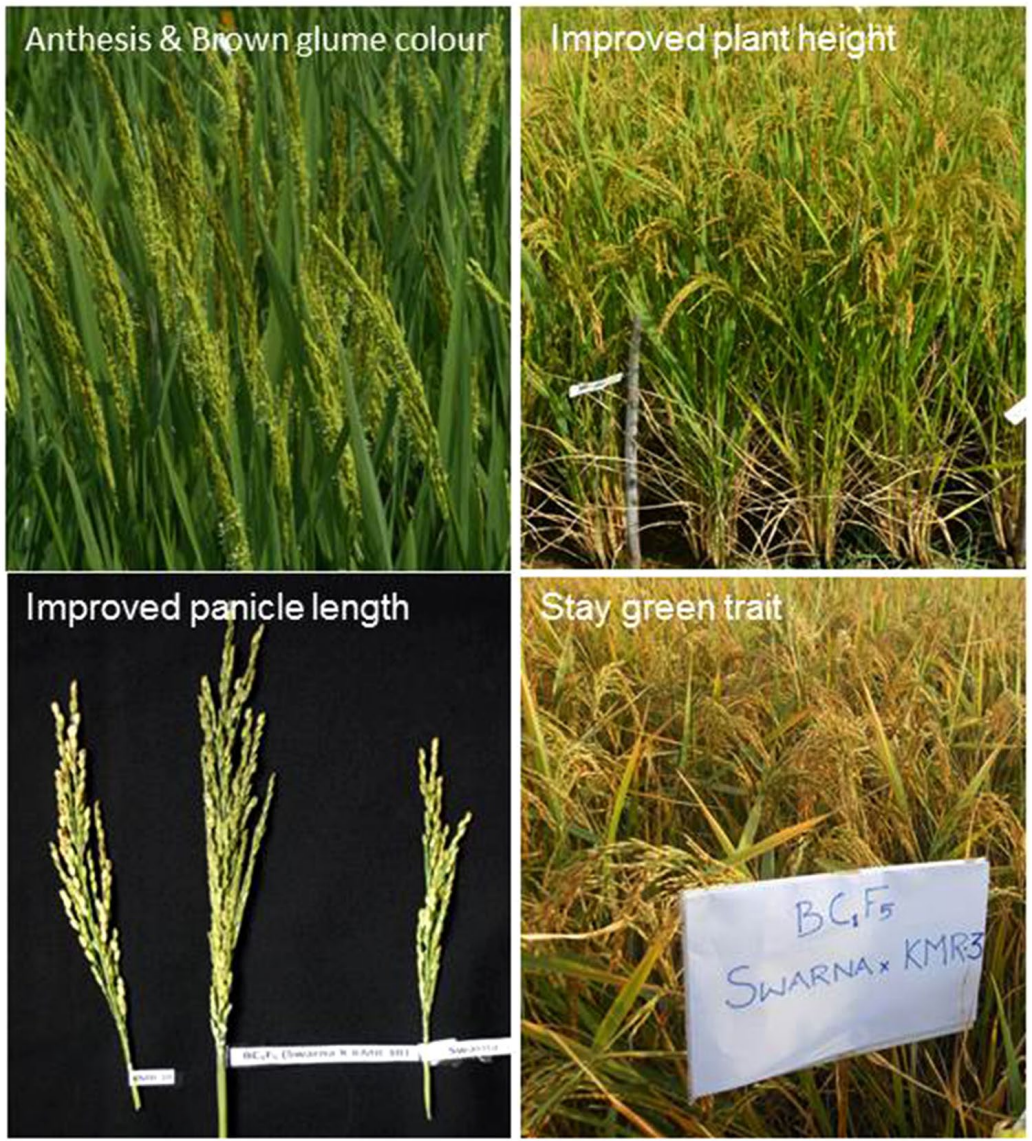
Converted restorers with improved plant height, panicle length with Swarna’s specific traits of stay greenness, golden hull colour.

To the best of our knowledge, the present study is the first attempt through MABB approach for converting partial restorer Swarna into complete restorer by transferring *Rf3* gene. We could recover Swarna genome in the backcross derived lines of converted restorers of BC_1_F_5_ and BC_2_F_4_ generations by 80% and 92.3%, respectively. The agro morphological evaluation of converted restorers (RP5934-66 to RP 5934-100) in the background of Swarna exhibited desirable traits for an ideal restorer lines viz., improved plant height, longer panicles, more number of productive tillers with heavy pollen load along with traits specific for Swarna, like brown glume or golden husk colour with stay green type etc (Fig. 6). The restorers should be ideally be taller than that of their CMS line for easy and higher hybrid seed production, as taller restorer parents facilitates easy pollen deposition on the stigma lobes of CMS lines. In our study BC_1_F_5_ lines were preferred as restorers than BC_2_F_4_ lines because of desirable plant height. BC_2_F_4_ lines expressed shorter plant height similar to the recurrent parent Swarna. We were able achieve target trait of complete fertility restoration in BC_1_F_5_ generation by restricted marker assisted back cross breeding involving only single back cross followed by selfing and selection. To retain some of the useful traits from donor parent^29^, restricted marker assisted backcross breeding strategy has been used for improving basmati rice variety, Pusa Basmati 1 for bacterial blight resistance.

Longer panicle with more number of grains is also preferred traits of restorer for supplying more pollen grains for hybrid seed production. More number of productive tillers results in higher pollen shedding potential per sq.m. during peak anthesis period. Apart from restoration ability converted restorers were observed to be high yielding than Swarna indicating potentiality of these lines for releasing it as variety. The test crosses evaluation of F_1_ hybrids based on pollen and spikelet percentage confirmed that Swarna is a partial restorer and KMR3R is a complete restorer. The partial restorer Swarna possessing only *Rf*4 gene could restore the fertility of WA-CMS lines with an average of 70% pollen and spikelet fertility. This level of pollen and spikelet fertility is not sufficient warrant deployment of Swarna as an effective restorer in hybrid rice breeding. The effective restorers are expected to have more than 90% of pollen and spikelet fertility. The hybrids produced using converted Swarna breeding lines with *Rf3* gene showed 15–20% of more pollen and spikelet fertility than the partial restorer Swarna. This clearly demonstrates that that *Rf3* gene may be contributing 15 to 30% for the fertility restoration trait of WA-CMS and *Rf*4 may be contributing about 70% for the same trait and presence of both the genes can bestow complete fertility restoration^13,33–38^.

An effective restorer, IR64 carrying both *Rf4* and *Rf*3 genes showed 85.4% spikelet fertility and Basmati 370 partial restorer carrying only *Rf4* gene showed 59.3% of spikelet fertility explaining that presence of both the *Rf* genes will enhance pollen and spikelet fertility percentage^19^. The results obtained from the present study confirms that presence of only *Rf4* gene in restorers may result in partial restoration of fertility in hybrids and presence of both *Rf4* and *Rf*3 genes will certainly results in complete restoration. The rice varieties having both the restorer genes expressing higher fertility restoration has been reported^11,39^. The restorer lines carrying two dominant genes for fertility restoration should be preferred to develop rice hybrids using WA-CMS lines and hence the resultant hybrids are expected to have higher fertility and thereby higher heterosis^40,41^.

As cited above most of the previous reports were based only on molecular screening for the presence of *Rf4* and *Rf*3. Our study is the first instance of using marker-assisted selection for targeted introgression of fertility restorer gene(s) in order to develop improved parental lines in the background of an elite Indian mega- variety of rice and thereby confirming the major and minor role of *Rf4* and *Rf*3. The grain quality characters of the converted (i.e. improved) restorer lines in the genetic background of Swarna were similar to that of the original parent. In a panel test for grain quality traits conducted at ICAR-IIRR, Hyderabad, it was observed that, the *Rf*3 gene introgressed lines were indistinguishable from Swarna in terms of color of paddy, grain size and shape. Further, replicated field trials that were carried out at twelve different locations across India under the all India coordinated rice improvement project (AICRIP), showed that yield levels of the improved lines were significantly higher than Swarna and the check lines indicating that there is no apparent yield penalty associated with presence of the *Rf*3 gene^3^. Considering these points, improved version of restorers developed in the genetic background of Swarna would be of great use in hybrid rice breeding for developing late duration hybrids. Large scale seed production of potential hybrids involving long duration improved restorers with different WA-CMS lines are under pipeline for multi location testing. Some of the improved lines were also demonstrated their potentiality as higher heterotic hybrids in the early and medium duration category (Tables 5a,b).

Introgression of fertility restorer genes without recovery of other characters of recurrent parent would have been a futile exercise, if the developed lines do not meet expected grain and cooking quality with higher yields. We feel that this was made possible because of the extensive phenotypic selection and also because of the use of molecular markers for background selection. The selected BC_1_F_5_ and BC_2_F_4_ lines possessed the desirable traits of restorers like intermediate or tall plant height, early to late duration, heavy pollen load, optimum productive tillers and long panicles (Fig. 6). This was achieved by stringent phenotypic selections in each backcross generations as demonstrated in the study^42^. As indicated earlier, two rice hybrids namely IIRRH-111 and IIRRH-112 developed by using these improved lines have undergone multi location testing under the All India Coordinated Rice Improvement Project-Initial hybrid rice trial during *kharif* 2016. The rice hybrid IIRRH-112 was found promising with yield advantage of 10% superiority over hybrid and varietal checks in zone III (eastern), whereas Swarna is a very popular rice variety and occupying major area under cultivation.

To the best of our knowledge, this is the first attempt to convert partial restorer to complete restorer by transferring *Rf* genes through marker-assisted backcross breeding. Thus, in the present study, we have successfully developed restorers with characteristic features of popular mega Indian rice variety Swarna for utilization in hybrid rice breeding, specially for development of late maturity hybrids.

## Materials and Methods

The experimental materials consist of Swarna (MTU 7029) a popular mega rice variety, which occupies larger area under cultivation in India, derived from the cross Vasistha x Mahsuri and is a partial restorer and it was utilized as the recurrent parent for improvement of its fertility restoration ability. The donor parent for the fertility restoration trait/*Rf* gene(s) was KMR3R (Jaya/IR29723-143) a restorer parent of popular rice hybrid KRH-2. KMR3R has been identified to carry both fertility restorer genes *Rf*_*4*_ and *Rf*_3_ genes^18^. To determine the fertility restoration status of recurrent and donor parent, crosses were made with four CMS lines namely APMS 6A, IR 79156A, IR 68897A and IR 58025A to produce F_1_ hybrids during *rabi* 2011. These F_1_ hybrids were evaluated for their pollen and spikelet fertility and grain yield heterosis during two rice crop seasons viz., *kharif* 2012 (i.e. wet season 2012) and *rabi* 2013 (i.e. dry season 2013) to confirm their fertility restoration status.

### Pollen fertility

Pollen fertility was measured using anthers collected from the spikelets at one or two days before anthesis. The anthers from each spikelet were smeared in a drop of 1% Iodine-potassium iodide (I_2_-KI) solution^43^, on a glass slide and three randomly selected microscopic fields were counted. Stained, well filled and round pollen grains were counted as fertile, while unstained, shrivelled and empty pollen grains were considered as sterile. Pollen fertility was calculated and expressed in percentage as given below

$${\rm{Pollen}}\,{\rm{fertility}}\,({\rm{PF}}) \% =\frac{{\rm{Total}}\,{\rm{number}}\,{\rm{of}}\,{\rm{stained}}\,{\rm{pollen}}\,{\rm{grains}}}{{\rm{Total}}\,{\rm{number}}\,{\rm{of}}\,{\rm{pollen}}\,{\rm{grains}}\,{\rm{examined}}}\times 100$$

Further the plants were classified into the following classes, fertile (more than 80%), partially fertile (51–80%), partially sterile (21–50%) and completely sterile (0–20%)

### Spikelet fertility

The panicles that emerged from the primary tiller were bagged before anthesis to avoid out crossing and the number of filled grains and chaffs in the panicle were counted at the time of maturity. The ratio of filled grains to the total number of spikelets was expressed as spikelet fertility as given below:

$${\rm{Spikelets}}\,{\rm{fertility}}\,({\rm{SPF}}) \% =\frac{{\rm{Number}}\,{\rm{of}}\,{\rm{filled}}\,{\rm{spikelets}}\,{\rm{in}}\,{\rm{the}}\,{\rm{panicle}}}{{\rm{Total}}\,{\rm{number}}\,{\rm{of}}\,{\rm{spikelets}}\,{\rm{in}}\,{\rm{the}}\,{\rm{panicle}}}\times 100$$

Plants were classified into four classes based on spikelet fertility percentage, namely, fertile (more than 85% spikelet fertility), partially fertile (50–85%), partially sterile (21–50%) and completely sterile (0–20%)

### Molecular screening for the presence of *Rf* genes

#### DNA isolation and PCR conditions

The total genomic DNA was isolated from the 20 days old transplanted seedlings according to the procedure reported by^44^. PCR analysis was carried out using 30–50 ng DNA as template, 5 pmoles of each primer, 0.05 mM dNTPs, 10X PCR buffer (TaKaRa Taq™ DNA Polymerase) and 1 U Taq DNA polymerase in a total volume of 10 µl. Template DNA was initially denatured at 94 °C for 5 min followed by 35 cycles of PCR amplification with the following parameters: a 30 s denaturation at 94 °C, a 30 s annealing at 55 °C and 1 min of primer extension at 72 °C. A final extension was done at 72 °C for 7 min. The amplified products were electrophoretically resolved on a 3% agarose gel containing 0.5 µg/ml of ethidium bromide in 0.5X TBE buffer and visualized under UV and results were documented. The recurrent parent Swarna, donor parent KMR 3 R along with few popular varieties (partial restorers) were screened with 20 reported SSR markers linked to *Rf4* & *Rf*3 genes to identify polymorphic marker between restorer and partial restorers for foreground selection (Table 2) and for background selection microsatellite and InDel markers that are polymorphic between donor and recurrent parents were identified by screening 728 markers distributed throughout the rice genome (Table 3). Using the data from polymorphic SSR markers, a schematic map illustrating the genomic contribution of donor and recurrent parents was prepared using Graphical Genotype (GGT) Version 2.0^45^ to identify backcross derived lines possessing maximum recurrent parent genome. The parental polymorphic markers were used to genotype, positive foreground selected plants at each backcross generation to estimate the amount of recurrent parent genome contribution ‘G’ which was calculated as per the following formula:

$${\rm{G}}=[({\rm{X}}+1/2{\rm{Y}})\times 100]/{\rm{N}}$$

where,

N = total number of parental polymorphic markers screened

X = number of markers showing homozygosity for recurrent parent allele

Y = number of markers showing heterozygosity for parental alleles.

### Marker-assisted backcross breeding (MABB)

**Figure 7:**
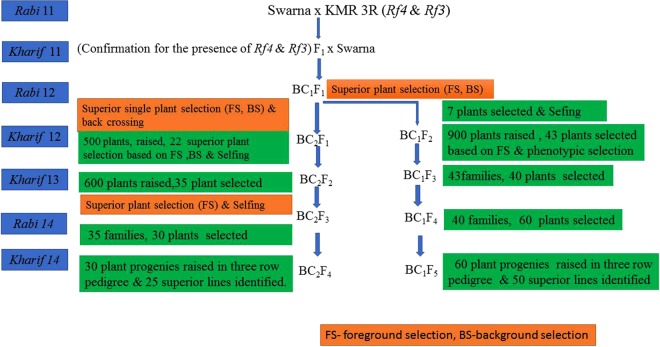
Schematic workflow of marker assisted backcross breeding of Swarna x KMR3R.

A cross was made between Swarna x KMR3R to improve the fertility restoration trait of the recurrent parent Swarna, during *rabi* 2011 (i.e. dry season 2011). The backcross breeding procedure followed is presented in the Fig. 7. The F_1_ plants of Swarna x KMR3R were screened using DRRM-RF3-10 identified polymorphic SSR markers between donor and recurrent parent. The true F_1_s were then backcrossed with Swarna to generate BC_1_F_1_s, which were confirmed for the presence of fertility restorer gene(s) *Rf*3 and *Rf4* with the help of DRRM-RF3-10 and PPR3 markers (i.e. foreground selection). The plants which were positive for the restorer genes *Rf4* and *Rf*3 were subjected to background selection with a set of 89 identified polymorphic SSR markers to identify a single BC_1_F_1_ plant, possessing maximum recovery of the recurrent parent genome (RPG). This plant was selfed to generate BC_1_F_2_s and also backcrossed with Swarna to generate BC_2_F_1_s. The MABB process involving foreground and background selection as explained above was repeated among the BC_2_F_1_ plants and the best BC_2_F_1_ plant was selfed to generate BC_2_F_2_ seeds. The BC_1_F_2_ and BC_2_F_2_ were analyzed with markers specific for *Rf*3 and *Rf4* to identity homozygous plants. Homozygous BC_1_F_2_ and BC_2_F_2_ plants were then advanced through pedigree method of selection for further phenotypic evaluation based on duration of flowering (days), plant height (cm), number of tillers, panicle length (cm) and single plant yield (g). Phenotypically superior plants were advanced for further evaluation.

### Evaluation of agro-morphological characters of the backcross derived lines

Thirty days old seedlings at BC_1_F_5_ and BC_2_F_4_ generations along with the parents were transplanted in the main field with the spacing of 20 × 20 cm and fertilizer dosage of 120:80:60 (N:P:K) kg/ha during *kharif* 2014. The experimental plots were arranged in alpha lattice design in four blocks with two replications. Standard agronomic practices were followed while raising the rice crop. Data was recorded on randomly selected five plants in each replication for the agronomic traits, viz. flowering duration, plant height (cm), number of productive tillers, panicle length (cm), grain yield per plant, and 1000-grain weight. The plants were visually observed for the following traits viz., heavy pollen load during anthesis, strong culm, non-lodging type and Swarna’s specific traits of stay greenness and golden hull colour for selecting superior phenotypic plants as restorers. The data was tabulated and statistically analyzed using standard Microsoft office excel and SPSS package.

### Generation of experimental rice hybrids utilizing improved restorers and their evaluation

The phenotypically superior lines from BC_1_F_5_ and BC_2_F_4_ generations possessing *Rf4* and *Rf3* genes were crossed with two CMS lines viz., APMS 6A and CRMS 32A to produce F_1_ hybrids during *kharif* 2014 (i.e. wet season 2014). Thirty days old seedlings of experimental hybrids along with checks were transplanted to the field with the spacing of 20 × 20 cm in two replications. Data on days to flowering, plant height, productive tillers, panicle length, pollen and spikelet fertility percentage, grain yield and grain yield heterosis were estimated. Seeds of Swarna and selected experimental hybrids were stored for three months after harvesting and grain quality tests were carried out by standard grain quality evaluation protocols (as explained in^30^) with the rice grains having 12 to 14% moisture content. Superior rice hybrids were identified to produce hybrid seed in larger plots during *rabi* 2016 (i.e. dry season 2016) and nominated to multi-location evaluation of AICRIP trials during *kharif* 2016 (i.e. wet season 2016).
